# Distribution and Effects of Model Palladium-Doped Nanoplastics in the Marine Mussel *Mytilus galloprovincialis*

**DOI:** 10.3390/nano16181133

**Published:** 2026-09-10

**Authors:** Nagore González-Soto, Gabriella F. Schirinzi, Guillaume Bucher, Eider Bilbao, Amaia Orbea, Dora Mehn, Douglas Gilliland, Miguel-Ángel Serra, Denise M. Mitrano, Marisa Sárria Pereira de Passos, Miren P. Cajaraville

**Affiliations:** 1 CBET+ Research Group, Department Zoology and Animal Cell Biology, Faculty of Science and Technology and Research Centre for Experimental Marine Biology and Biotechnology PiE, University of the Basque Country EHU, 48620 Plentzia, Basque Country, Spain; nagore.gonzalez@ehu.eus (N.G.-S.); amaia.orbea@ehu.eus (A.O.); 2 Oceanic and Continental Environments and Paleoenvironments (EPOC UMR) 5805, University of Bordeaux, National Center for Scientific Research (CNRS), Bordeaux INP, 33600 Pessac, France; 3 European Commission, Joint Research Centre (JRC), 21027 Ispra, Italydora.mehn@ec.europa.eu (D.M.); douglas.gilliland@ec.europa.eu (D.G.); m.sarria@biotec.rwth-aachen.de (M.S.P.d.P.); 4 Department of Environmental Systems Science, Swiss Federal Institute of Technology Zurich (ETH), Universitätstrasse 16, 8092 Zurich, Switzerland

**Keywords:** palladium-doped polystyrene nanoplastics, foodborne exposure, mussels, hemocyte responses, histopathology, condition index

## Abstract

While nanoplastics (NPs, <1 µm) concentration is anticipated to increase in the marine environment, the analytical tools for NP quantification and data on their impacts are still scarce. This work aimed to expand knowledge regarding bioaccumulation and effects of NPs on the sentinel organism *Mytilus galloprovincialis*. For this, mussels were dietarily exposed for 7 days to model palladium-doped-polystyrene NPs (Pd-PS NPs) of 142.6 ± 1.0 nm at 1 × 10^9^ Pd-PS NPs/mL. ICP-MS analysis on water samples showed that after one day of exposure no Pd was detected suggesting that mussels cleared the NPs. After 7 days of exposure, 14% of the Pd introduced in the tanks was found in feces. Less than 0.02% passed into the hemolymph of mussels and consequently, no responses were observed on hemocytes. Approximately 1% of the Pd was found in soft tissues. This could be linked to the significantly higher prevalence of digestive tubule atrophy observed in exposed mussels in comparison to controls. No effects were observed at the whole organism level. In conclusion, mussels were able to uptake and to eliminate Pd-PS NPs through feces. Whether longer exposures could cause a significant accumulation of NPs and produce associated biological effects remains to be studied.

## 1. Introduction

Plastic contamination is a cause of increasing public concern [1]. Once in the marine environment, large plastics tend to fragment into smaller pieces, giving rise to plastic particles of continuously decreasing size [2]. Even though there is still debate on the definition of the smaller plastic particles [3], plastics between 1 µm and 1 mm are considered microplastics (MPs), and those below 1 μm nanoplastics (NPs) [4]. In fact, plastic particles smaller than 1 µm show a completely different behavior compared to larger ones and, therefore, these need to be considered in a different category [5].

Many studies have been conducted on MPs in recent years [6,7]. Similarly, an increasing number of studies are being conducted on NPs [8,9,10]. However, there are still multiple gaps in knowledge that need to be addressed in both MPs and NPs research [11]. These gaps stem from two major issues: the lack of standardization in research methodologies within the field, which makes comparisons among studies exceedingly difficult; and the lack of robust analytical methods for detecting the smallest particles, particularly at the nanometric size [1]. Analytical techniques developed during the last years for engineered nanomaterials and for MPs have been shown to be inadequate for direct application to NP detection and characterization [11,12].

Due to this lack of analytical methods, there is insufficient information available to conduct a thorough risk assessment of NPs [12], in terms of both hazard and exposure assessment. A major difficulty is establishing the actual concentration of NPs in the environment [4,11,13]. Recent works have reported the detection of NPs in seawater [4,5], but generally the concentration of NPs in the marine environment has been estimated using in silico models and simulation laboratory work [8]. Recently, Hietbrink et al. [14] reported for the first time the concentration of NPs (only taking into account polyethylene terephthalate, polystyrene (PS), and polyvinyl chloride) in North Atlantic water samples. They found between 1.5 and 32.0 mg/m^3^ of NPs and, based on these data, they estimated that the total NP mass in the North Atlantic gyre could be as high as 15.20 Mt. This value is 10 to 100 times higher than what has been estimated for MPs and macroplastics, respectively, thus indicating that NPs comprise the dominant fraction of marine plastic pollution [14].

Similarly, there are few works reporting the presence of NPs in marine organisms because detection limits of the techniques generally used for MPs identification, such as vibrational spectroscopies (Fourier Transform Infrared and Raman spectroscopies), are out of the nano range [15]. The few works that report data on the occurrence of NPs in marine biota are mostly based on techniques such as pyrolysis followed by gas chromatography/mass spectrometry (GC/MS) [16,17,18]. However, these techniques do not allow the determination of the cell and tissue distribution of NPs in marine organisms [19]. Studies reporting both the distribution of the particles in the target organisms and their effects are therefore needed to elucidate the potential hazards that NPs may pose in the marine environment [20].

For this aim, bivalves, and more precisely mussels, have been proposed as key organisms to study the toxicity of NPs [21]. Mussels are widely used in ecotoxicological studies due to multiple characteristics, e.g., they are easy to handle in the laboratory, they can be found worldwide, they are already used as bioindicators and sentinel species of pollution in the marine environment, and they have relative tolerance to pollutants even if they react to them [22]. Additionally, mussels are part of different trophic chains in marine ecosystems, besides being an economically relevant species, since they are used for human consumption. However, scientific data regarding the uptake, cell and tissue distribution and toxicological effects of NPs on bivalves are still scarce [23,24].

In laboratory experiments, it is possible to use labeled NPs to correlate NP uptake and distribution with toxicological effects. Among the different options available, radio-labeled particles are not commonly used for safety reasons [25]. On the other hand, fluorescently labeled particles have been widely used in recent years [26,27,28]. However, the concentration of particles needed to be able to detect some fluorochromes can be outside what is expected to be the environmentally relevant range [29]. In addition, fluorescently labeled particles may lose their fluorochromes. Furthermore, some cells and tissues of aquatic organisms have autofluorescence themselves, which may lead to misleading conclusions [30]. Metal-doped NPs appear to be a promising alternative. Specifically, palladium-doped PS NPs (Pd-PS NPs) have been used in recent studies for tracing the distribution of NPs in aquatic organisms by Inductively Coupled Plasma Mass Spectrometry (ICP-MS) of Pd [31,32,33,34]. Pd is scarcely found in the environment; it is not bioavailable in all of its forms and shows low toxicity [34,35]. In fact, the concentration of Pd encountered in water bodies and biota is in the nanogram range [36], ensuring that background levels in the environment will not interfere with laboratory exposure experiments.

In this context, this work aimed to expand knowledge regarding the potential distribution and effects of NPs on the sentinel marine mussel *Mytilus galloprovincialis*. For this, mussels were dietarily exposed to Pd-PS NPs for 7 days, and a battery of responses at different biological organization levels was analyzed, together with the presence of Pd-PS NPs in mussels’ soft tissues, hemolymph and feces. Furthermore, the interaction between the Pd-PS NPs and microalgae that was used as food was also studied.

## 2. Materials and Methods

### 2.1. Model Palladium-Doped Polystyrene NPs (Pd-PS NPs) Production and Characterization

Pd-PS NPs consisting of a polyacrylonitrile (PAN) core surrounded by a PS shell were synthesized following the protocol of Mitrano et al. [37]. Raman spectroscopic analysis was performed with a WITec alpha300 confocal Raman microscope (WITec GmbH, Ulm, Germany) equipped with a 532 nm laser, using a 10x objective. A drop of a concentrated particle suspension was deposited on a clean silicon surface and after drying of the drop, 10 spectra were collected with a 1 s accumulation time. After averaging and baseline subtraction, the spectrum was compared to that of pure PS and PAN. Raman spectroscopic analysis of the particles confirmed that the main component is PAN and that PS is a minor component, but spectral features such as the presence of the peak above 3000 cm^−1^ (aromatic ring C-H stretch) and around 1600 cm^−1^ (aromatic ring skeletal stretch) confirm the presence of PS in the particles.

A preliminary small-scale test was conducted prior to the major experiment, and therefore, two batches of Pd-PS NPs were produced and systematically characterized using different instrumental analyses (Table S1). No significant differences in morphology (raspberry shape, Figure S1) or in the physical properties (including particle density) were found among the two Pd-PS NPs batches synthesized (see Table S1).

ICP-MS analysis showed that the actual concentration of both stock suspensions was 10^13^ particles mL^−1^. Specifically, the Pd-PS NPs used in the large-scale (major) experiment demonstrated a size of 142.6 ± 1.0 nm and a negative surface net charge (−36.2 ± 4.7 mV) at pH 6.59. The actual concentration in mass of this stock measured by dry weight was 41.6 ± 0.39 g L^−1^, and the metal (Pd) content quantified by ICP-MS was 130.2 ± 10.5 mg L^−1^, representing approximately 0.3% of the total mass. The structure of these NPs guarantees that the Pd remains stable in the core of the particles and ensures minimal leaching over the course of the experiments, as previously reported under different conditions [37,38,39,40,41].

#### 2.1.1. Size Distribution and Density Characterization by Centrifugal Sedimentation Methods

Centrifugal sedimentation methods (analytical ultracentrifugation and line start disk centrifugation) were used to characterize the size distribution and density of the Pd-PS NPs. Both methods determine particle size distribution by measuring the sedimentation speed of particles and calculate particle size considering the Stokes equation [42], which requires particle density as an input value. If particle density is not known, multiple measurements are needed at various liquid densities to determine it (ISO 18747-2:2019) [43] as well. Line start disk centrifugation was further considered to assess the Pd-PS NPs’ propensity to aggregate and/or agglomerate in artificial saltwater (38 practical salinity units, PSU; pH 8.16 at 19.7 °C).

##### Analytical Ultracentrifugation

A Proteomelab XL-I analytical ultracentrifuge (Beckman Coulter, Brea, CA, USA) equipped with interference and absorbance optics was used to register the sedimentation of the particles in 10% and 20% aqueous sucrose solution at 5000 rpm rotation speed in two-sector sapphire window cells. The sample sector was loaded with 400 µL of 100× diluted sample in 10% or 20% sucrose, while the reference cell contained 390 µL of the corresponding sucrose solution. Interference data were fit using the ls-g*(s) model of the software sedfit with a linear grid in the 0–20,000 S range at 200 resolution. The modes of the resulting sedimentation coefficient distributions were inserted as sedimentation speed values in the equation suggested by the multi-velocity approach (ISO 18747-2:2019) [43]. For 10% sucrose, density and viscosity values of 1.038 g cm^−3^ and 1.325 mPa s, and for 20% sucrose, values of 1.078 g cm^−3^ and 1.902 mPa s were applied.

##### Line Start Disk Centrifugation

In order to determine the realistic size distribution of the particles in artificial saltwater, experiments with a line start disk centrifuge were performed using a CPS DC24000 UHR disk centrifuge (CPS Instruments, Inc., Oosterhout, The Netherlands) in two different sucrose density gradients: 0–8% and 8–24% at 22,000 rpm rotation speed. As seawater has higher density (1.0236 g cm^−3^ at 25 °C) than ultrapure water (0.9970 g cm^−3^ at 25 °C), the sucrose gradient was also prepared in the artificial saltwater. This also allowed maintaining the salinity of the medium during the full sedimentation process. Refractive index and absorption values of 1.59 and 0.001, respectively, were inserted for the particles. Before each measurement, calibration with 237 nm polyvinyl chloride size standard (CPS Instruments, Inc., Oosterhout, Netherlands) was performed. Owing to the calibration, the resulting size distributions are not sensitive to the density and viscosity of the gradient; thus, average values estimated based on 0–8% and 8–24% sucrose gradients in ultrapure water (18.2 MΩ cm at 25 °C) were applied. After calibration, an aliquot of 100 µL of 100× diluted particles in artificial saltwater was injected into the instrument using a 1 mL syringe. Experiments were performed in triplicate. Mass-weighted size distributions determined in the two gradients were recalculated using various particle density values to find the density providing the best match between the modes of the size distributions.

### 2.2. Experimental Design

Roughly 70 marine mussels, *Mytilus galloprovincialis* (3.5–5.5 cm shell length), were collected at Pobra do Caramiñal (42°36′03.8″ N, 8°55′34.9″ W, Galicia, Spain) in September 2023 and supplied by Angulas Aguinaga S.A.U. (Irura, Basque Country, Spain). Mussels were placed in a single large propylene tank (600 L) with a recirculating seawater system and left without feeding for two days. Seawater collected at the mouth of the Butroi estuary (43°24′47.0″ N, 2°56′52.8″ W) was naturally filtered by sand in the uptake wells, aided by a pump that sent the water to the Plentzia Marine Station (PiE-EHU). Seawater gas balance was controlled at the station and then passed through a decantation/inertial tank and filtered (mesh size ≤ 3 μm). Starting on the third day, mussels were fed once per day with the marine microalgae *Isochrysis galbana* (T-iso clon) (2 × 10^7^ cells mussel^−1^ day^−1^) for 3 days. *I. galbana* was obtained from a clean, high-quality microalgae stock from the Spanish National Algae Bank through a commercial dealer (Power Aquaculture S.L, Madrid, Spain) and grown at the Marine Station microalgae culture room at 19 °C, constant light (36 W), and fed with F/2 Algae Food (Fritz Pro Aquatics, Mesquite, Nevada). During acclimation of mussels, the light regime was 12 L/12 D, and room temperature was kept constant at 16 °C. Water parameters (conductivity, dissolved O_2_, pH, and temperature) were monitored daily with a multichannel probe. Water parameters were as follows: temperature: 20.06 ± 0.34 °C, dissolved O_2_ > 80%, conductivity: 32.95 ± 0.10 PSU, and pH: 7.51 ± 0.47.

Two experimental groups were established: control and dietarily exposed to Pd-PS NPs. Each condition was run in duplicate. Following the preliminary experiment (Text S1), NPs water exposure concentration of 10^9^ particles mL^−1^ (1.91 mg L^−1^ of NPs, 5.97 µg L^−1^ of Pd) was established to be above the limit of detection of Pd by ICP-MS (0.1 ng g^−1^) in target samples. This concentration in water could result in 5.88 × 10^11^ Pd-PS NPs mussel^−1^ day^−1^.

**Figure 1:**
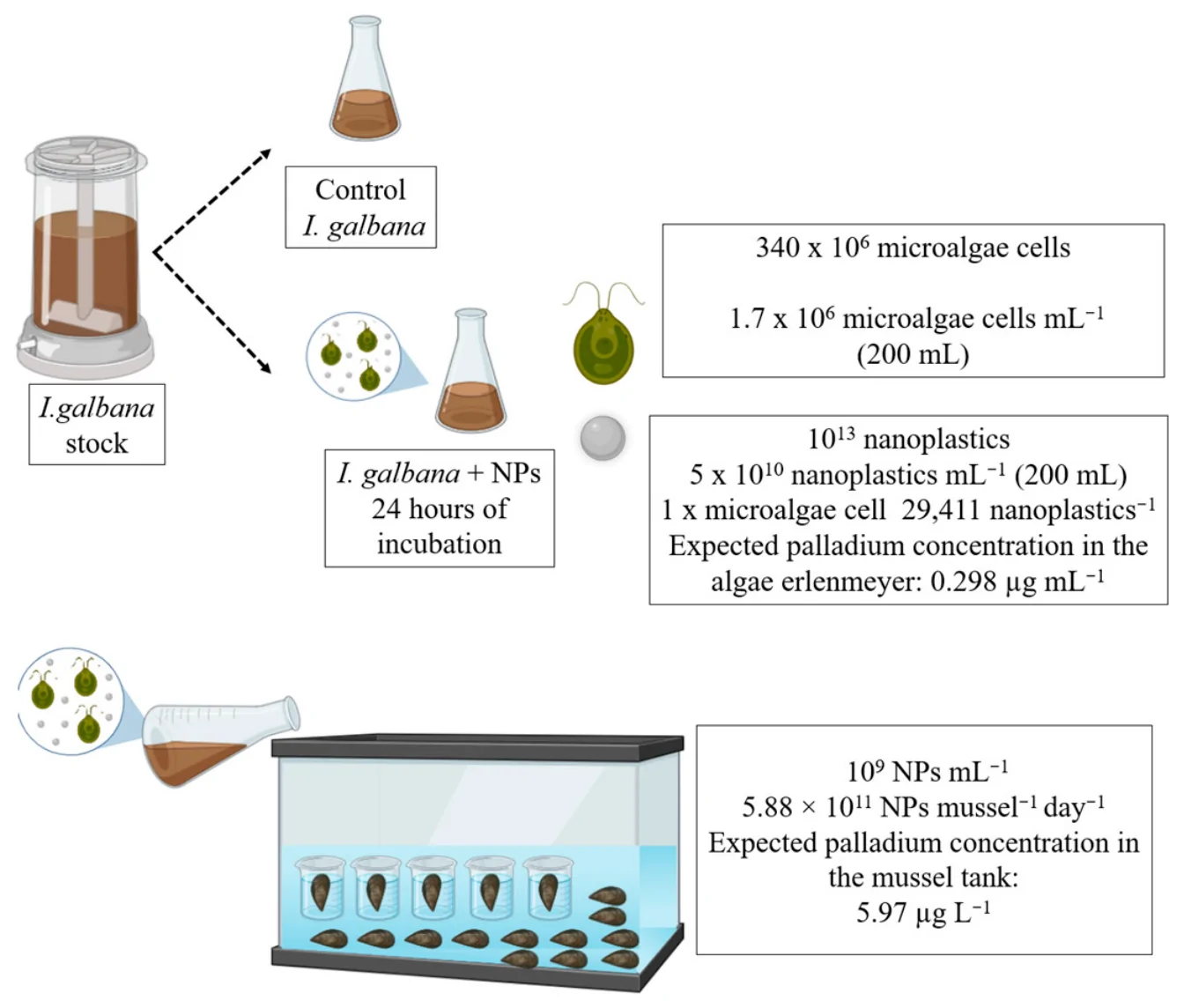
Schematic representation of the experimental foodborne exposure of marine mussels *Mytilus galloprovincialis* to Pd-PS NPs using *I. galbana* microalgae.

After 5 days of acclimation, 17 mussels were placed in 30 L aquaria (filled to 10 L); 5 of them per aquarium were placed individually within 100 mL glass beakers to enable the collection of their feces. Mussel feces were collected on days 1, 3, and 7 of exposure to analyze the Pd-PS NPs content. On days 2, 4, 5, and 6 of exposure, feces were also removed from the tanks before the renewal of water and nanoplastic dosing. In this way, the collected feces corresponded only to the day of feces collection. Every day, the whole water volume of the tank was renewed, and mussels were fed with *I. galbana* microalgae with (exposed) or without (control) NPs (Figure 1). For this, 4 aliquots of 3.4 × 10^8^ microalgae cells (2 × 10^7^ cells ∗ 17 mussels) were taken from the stock culture in a 0.5 L Erlenmeyer and were mixed with the desired concentration of NPs for 24 h (Figure 1). As the concentration of the stock algae culture varied from day to day, to avoid bias during the experiment, the mix of algae and NPs was brought to 200 mL with filtered seawater.

During exposure, the light regime and room temperature were kept at 12 L/12 D and 16 °C, respectively. Water parameters (conductivity, dissolved O_2_, pH, and temperature) were checked daily with a multichannel probe just after water renewal and before dosing of microalgae. Water parameters were as follows: temperature: 20.095 ± 0.34 °C, dissolved O_2_ > 80%, conductivity: 32.96 ± 0.12 PSU, and pH: 7.46 ± 0.51.

### 2.3. Determination of Pd in Different Matrices

ICP-MS analysis of Pd was carried out in the following matrices: saltwater, microalgae, mussels’ soft tissues, feces, and hemolymph.

Seawater samples (1 L) were collected at the beginning of the experiment (t0), after the first day of exposure (t1), and at the end of the experiment (t7) in one replicate tank. Samples were kept refrigerated at 4 °C until the end of the experiment. Then, each sample was individually concentrated using a 25 mm diameter 400 mL Amicon 8050 stirred cell (Millipore Co., Bedford, MA, USA), equipped with a membrane disk (Biomax^®^ polyethersulfone membrane, 76 mm, NMWL 300 kDa, Millipore Co., Bedford, MA, USA) to reach the final volume of 50 mL. Filters were then sonicated with the concentrate exposure medium before taking an aliquot for acidic digestion.

Samples of control and exposed microalgae were collected in each replicate tank for Pd-PS NPs content determination on days 1, 3, and 7 of exposure. All samples were frozen at −40 °C until analysis.

As mentioned above, 5 mussels per replicate tank were placed in beakers to individually collect the feces for Pd-PS NPs content determination. Mussel feces were collected on days 1, 3, and 7 of exposure and pooled by day and tank. All samples were kept frozen at −40 °C until analysis.

After 7 days of exposure, the hemolymph of 10 mussels per replicate tank was withdrawn from the posterior adductor muscle. Individual hemolymph samples were diluted with the same volume of antiaggregant solution (0.47 M NaCl + 1 mM EDTA, pH 7.5). Then, soft tissues were removed and placed in glass vials. Both the hemolymph and mussel flesh samples were frozen at −40 °C, and in the case of mussels’ soft tissues, these were then freeze-dried and ground into powder for Pd content analysis.

#### 2.3.1. Samples Pre-Treatment and Acidic Digestion Protocol

An acidic digestion step was added to ensure the complete removal of the organic content of the biomatrices and guarantee the breakdown of the PS shells for detection of Pd by ICP-MS analysis. Mussels’ soft tissues (200 mg, dry weight), feces (200 µL), microalgae (500 μL) and concentrated seawater (1 mL) were placed in the digestion vessels, and 0.5 mL of hydrogen peroxide (H_2_O_2_, 30% *w*/*w*, Sigma Aldrich, St. Louis, MO, USA) was added to assist the degradation of the organic matter. After 10 min, 4 mL of nitric acid (HNO_3_ ≥ 69%, Fluka, Seelze, Germany) was added, followed by microwave digestion using the CEM Diskover SP-D system (CEM Corp., Kamp Lintfort, Germany). In the digester, the following parameters were set: 240 °C, 5 min ramp time, 30 min hold time, and 300 W maximum power, followed by a cooldown time of roughly 7 min to 50 °C.

In the case of hemolymph samples, these were pooled (0.5 mL × 5) and incubated in tubes for 2 h with 0.5 mL of H_2_O_2_ (30% *w*/*w*, Sigma Aldrich) and 0.4 mL of HNO_3_ (≥69%, Fluka) using the Eppendorf ThermoMixer C (Hamburg, Germany), set at 80 °C. After cooling, the digested samples were diluted with ultrapure water (18.2 MΩ cm at 25 °C), resulting in a final nitric acid concentration of ~5% (*w*/*w*).

#### 2.3.2. ICP-MS Measurement

To determine the Pd content in the digested samples, the measurements were carried out with Agilent’s 7700 ICP-MS (Agilent Technologies, Waldbronn, Germany), equipped with a CETAC ASX-500 series autosampler (Omaha, NB, USA) and a MicroMist nebulizer coupled to a nickel sampler cone (Wayne, PA, USA). The Agilent MassHunter software package (Workstation Software, *v* B.01.01, Build 123.11, Patch 4, 2012) was used for data processing. The metal (Pd) content was monitored by tracking ^104^Pd, ^105^Pd, ^106^Pd, ^108^Pd, ^88^Sr, ^90^Zr, ^111^Cd, ^89^Y, and ^115^In. The instrument was tuned to optimize sensitivity and minimize oxides and double charges to <1%. It was subsequently calibrated using a Pd standard solution (Pd standard for ICP TraceCERT^®^, 1000 mg L^−1^ in 5% HCl, Sigma-Aldrich), with quality control checks performed to verify the accuracy of the results. Yttrium (^89^Y) and Indium (^115^In) at the nominal concentration of 1 μg L^−1^ were used as internal standards to compensate for any signal changes due to matrix potential residual contribution. Samples with unexpectedly high concentrations were diluted and re-analyzed, as were samples that followed the initial high-concentration sample. Results are given as µg L^−1^ with the limit of detection (LOD) and the limit of quantification (LOQ) of ^106^Pd at 0.05 and 0.16 µg L^−1^, respectively.

#### 2.3.3. Bioaccumulation Factor (BAF) and Mass Balance

The bioaccumulation factor (BAF) was calculated for exposed mussels at the end of the experiment, as suggested by Arnot & Gobas [44]:

$$\mathrm{BAF}\;(L\;{\mathrm{kg}}^{-1})=\frac{Total\;Pd\;concentration\;in\;mussel\;tissues\;(µg\;{kg}^{-1})}{Measured\;exposure\;concentration\;of\;Pd\;in\;water(µg\;L^{-1})}$$

Pd concentrations obtained after 7 days of exposure were used to estimate the Pd mass balance. The input was defined as the measured concentration of Pd in water after the first dosage. The outputs considered were as follows: the concentration of Pd measured in water at the end of the experiment (in mg L^−1^ multiplied × 10 L), the concentration of Pd in feces at day 7, and the concentrations of Pd in mussels’ hemolymph and soft tissues at day 7. The Pd concentrations measured in feces, hemolymph, and soft tissues in mg L^−1^ or mg g^−1^ were extrapolated to the total number of mussels in the tank.

### 2.4. Cellular Biomarkers in Mussels’ Hemocytes

After 7 days of exposure, hemolymph of 5 mussels per replicate tank was withdrawn from the posterior adductor muscle. The extracted hemolymph was kept individualized per mussel and diluted with a 1:1 volume of antiaggregant solution. Then, 100 µL of the hemolymph–antiaggregant mixture per well was immediately seeded in 96-well PS microplates. Hemocytes were allowed to attach in an incubator at 18 °C for 1 h before removing the serum from the hemolymph. Cell viability, reactive oxygen species (ROS) production and phagocytic activity assays were conducted thereafter. Cell viability was assessed by thiazolyl blue tetrazolium bromide (MTT) assay according to Katsumiti et al. [27]. Briefly, cells were incubated with MTT (0.5 mg mL^−1^) for 2.5 h at 18 °C in the dark. Medium was then completely removed from the wells and the resulting formazan product was extracted with dimethyl sulphoxide by pipetting until it was completely dissolved. The plate was shaken, and absorbance was read at 570 nm in a Cytation 5 multireader (BioTek, Winooski, VT, USA).

ROS production assay was performed according to Katsumiti et al. [27]. Here, cells were incubated with 200 µL of carboxy-H_2_DCFDA (25 µM) for 30 min at 18 °C in the dark. The medium was then removed and the cells were washed twice with phosphate-buffered saline (PBS) at pH 7.4. The fluorescence was measured (λ e_x_/e_m_ = 492/527 nm) using the Cytation 5 multireader.

The phagocytic activity was assessed by the Texas Red-labeled zymosan (Invitrogen, Waltham, MA, USA) phagocytosis assay, which was performed according to Duroudier et al. [45] with modifications. Shortly, cells were incubated with 50 µL of Texas Red-labeled zymosan (10^7^ particles mL^−1^) for 30 min at 18 °C in the dark. After washing with PBS pH 7.4 to remove all non-phagocytosed particles, cells were incubated with an acetic acid–ethanol solution to extract the fluorochrome. Finally, fluorescence (λ e_x_/e_m_ = 595/615 nm) was measured using the Cytation 5 multireader.

Total protein quantification was performed (Bradford assay) to normalize absorbance and fluorescence intensity data obtained in each assay per mg of protein (Bradford, [46]).

### 2.5. Gamete Development, Gonad Index and Histopathology of Gonad and Digestive Gland

The digestive glands and gonads of 5 mussels per tank were dissected and processed following a standard histology protocol. Briefly, tissues were fixed in 4% (*v*/*v*) formalin in individual cassettes and dehydrated through an ethanol series concluding in xylene using an automatic tissue processor (Leica ASP300; Leica Instruments, Wetzlar, Germany). Subsequently, samples were embedded in paraffin, and 5 μm sections were cut using a Leitz 1512 microtome (Leica Instruments). Slides were dried in an oven at 37 °C for 24 h, stained with hematoxylin/eosin using an autostainer XL V2.02 (Leica), mounted in DPX, and examined under a BX51 light microscope (Olympus, Tokyo, Japan). On digestive gland sections, the prevalence of inflammatory reactions, including hemocytic infiltration, brown cell accumulation in digestive tracts and connective tissues, and granulocytomas, was determined. The prevalence of other pathologies such as connective tissue fibrosis and necrosis and atrophy of digestive tubules was also assessed [10,47,48,49]. On gonad sections, the sex ratio, gamete developmental stages and gonad index (GI) were determined. Six gamete stages were distinguished [50] and a GI value, ranging from 0 (resting gonad) to 5 (mature gonad), was assigned to each developmental stage as in Ortiz-Zarragoitia et al. [51], adapted from Kim et al. [47]. Oocyte atresia, connective tissue fibrosis, hemocytic infiltration and aggregation of brown cells were also assessed in gonads, following Ortiz-Zarragoitia et al. [51]. In both tissues, the presence of parasites was also recorded [47,48,49,52].

The prevalence of each alteration (number of individuals showing each pathology divided by the number of individuals in each group) was calculated as a percentage. In addition, the intensity of each alteration was assessed using a semiquantitative scale: 0—normal tissue, 1—less than half of the tissue is affected, 2—about half of the tissue is affected, 3—more than half of the tissue is affected, and 4—the overall tissue is affected [47]. Intensity was calculated as Sp/NH, where Sp is the score corresponding to the intensity of the histopathological alteration, and NH is the number of specimens with the histopathological alteration [49]. Both for prevalence and intensity of oocyte atresia, only females were taken into consideration.

### 2.6. Whole Organism Responses: Condition Index

Mussels’ shells were measured (height, length, width) using a Vernier caliper and weighed (total weight and shell weight) for calculation of the condition index (CI). The CI of each mussel was determined as a ratio of tissue wet weight (g) to internal cavity volume (cm^3^), according to Lobel et al. [53], modified for wet weight by Mubiana et al. [54].

### 2.7. Data Analysis

When possible, outliers were eliminated with the Chauvenet method [55]. Statistical analyses were carried out with SPSS 24 (IBM Analytics, Armonk, NY, USA), and the significance level was established at *p* < 0.05. All data sets were tested for normality and homogeneity of variances using Kolmogorov–Smirnov’s and Levene’s tests, respectively. The normally distributed data, which met the assumptions of homogeneity of variances, were assessed by one-way ANOVA. When significant differences were found, Tukey’s post hoc test was conducted to identify significant differences among treatments. Data that did not meet the above-mentioned assumptions were analyzed using the non-parametric one-way Kruskal–Wallis test, followed by Dunn’s post hoc test when significant differences among groups were found. For histopathological data expressed as percentages, an *X*^2^ test was used. No differences were found between the two replicate tanks for the biological parameters; thus, data sets were mixed and displayed together. Data corresponding to the biological parameters measured per tank is provided in Table S4.

## 3. Results

### 3.1. Pd-PS NPs Characterization in Saltwater

Analytical ultracentrifugation (AUC) measurements in 10% and 20% sucrose provided sedimentation coefficients of 1510 S and 805 S, respectively. The density of the Pd-PS NP particles calculated by applying these values was determined as 1.209 g mL^−1^. Disk centrifuge-based measurements provided a particle density of about 1.215 g cm^−3^ in artificial saltwater. It is slightly higher, but still in line with the value determined by the multiple velocity method using AUC. The size distribution mean was found to be about 142.6 nm, and the size distribution showed the presence of smaller aggregates in artificial saltwater. Studies conducted using the line start disk centrifuge to assess the Pd-PS NPs propensity to aggregate and/or agglomerate in artificial saltwater permitted to clearly identify two sharp size distribution curves: one corresponding to a dominant size population of about 144.2 nm, also observed by SEM and TEM analysis (Figure S1); and another corresponding to a secondary size population of about 214.5 nm, which was close to the average size measured via dynamic light scattering (DLS). Lastly, Pd-PS NPs particle aggregates of about 301.8 nm were measured in artificial saltwater at the same time by a line start disk centrifuge. A value corroborated by DLS analysis, as an average size of 316.7 nm was detected.

### 3.2. ICP-MS Determination of Total-Pd Content in the Collected Matrices

#### 3.2.1. Water from the Exposure Tanks

No palladium was detected in the control water tank at any exposure time. Immediately after the first exposure, 5.57 µg L^−1^ of palladium was detected in the exposed mussel tank (93.3% of the nominal exposure dose), while no palladium was found at the end of the first 24 h of exposure (Table 1). At the end of the 7 days of exposure, 0.378 µg L^−1^ of palladium was detected in the exposed mussel tank, which corresponded to 6.33% of the daily nominal exposure dose and 0.90% of the whole experiment exposure dose (Table 1).

#### 3.2.2. Microalgae

**Figure 2:**
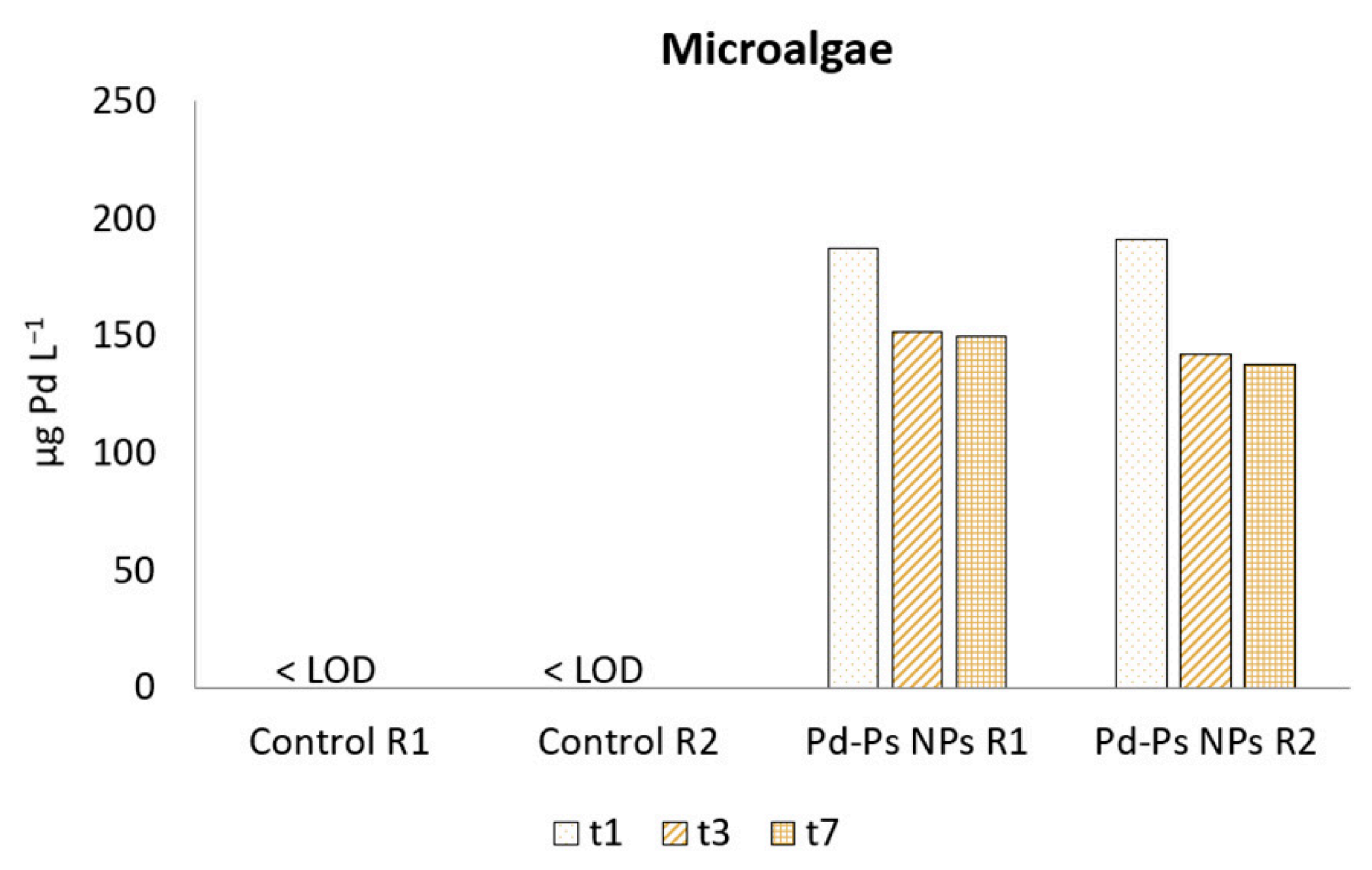
Concentration of Pd measured in microalgae over the exposure time (1, 3 and 7 days). Limit of detection (LOD): 0.05 µg L^−1^.

Looking at the palladium content of the microalgae cultures, no palladium was detected in the stock culture used to feed the control mussels (Figure 2). On the other hand, microalgae incubated with the Pd-PS NPs showed a similar palladium concentration over the exposure time, ranging from 189.4 ± 2.54 µg L^−1^ of palladium on day 1 of exposure to 143.8 ± 8.83 µg L^−1^ of palladium on day 7 of exposure (Figure 2).

#### 3.2.3. Mussels’ Feces

**Figure 3:**
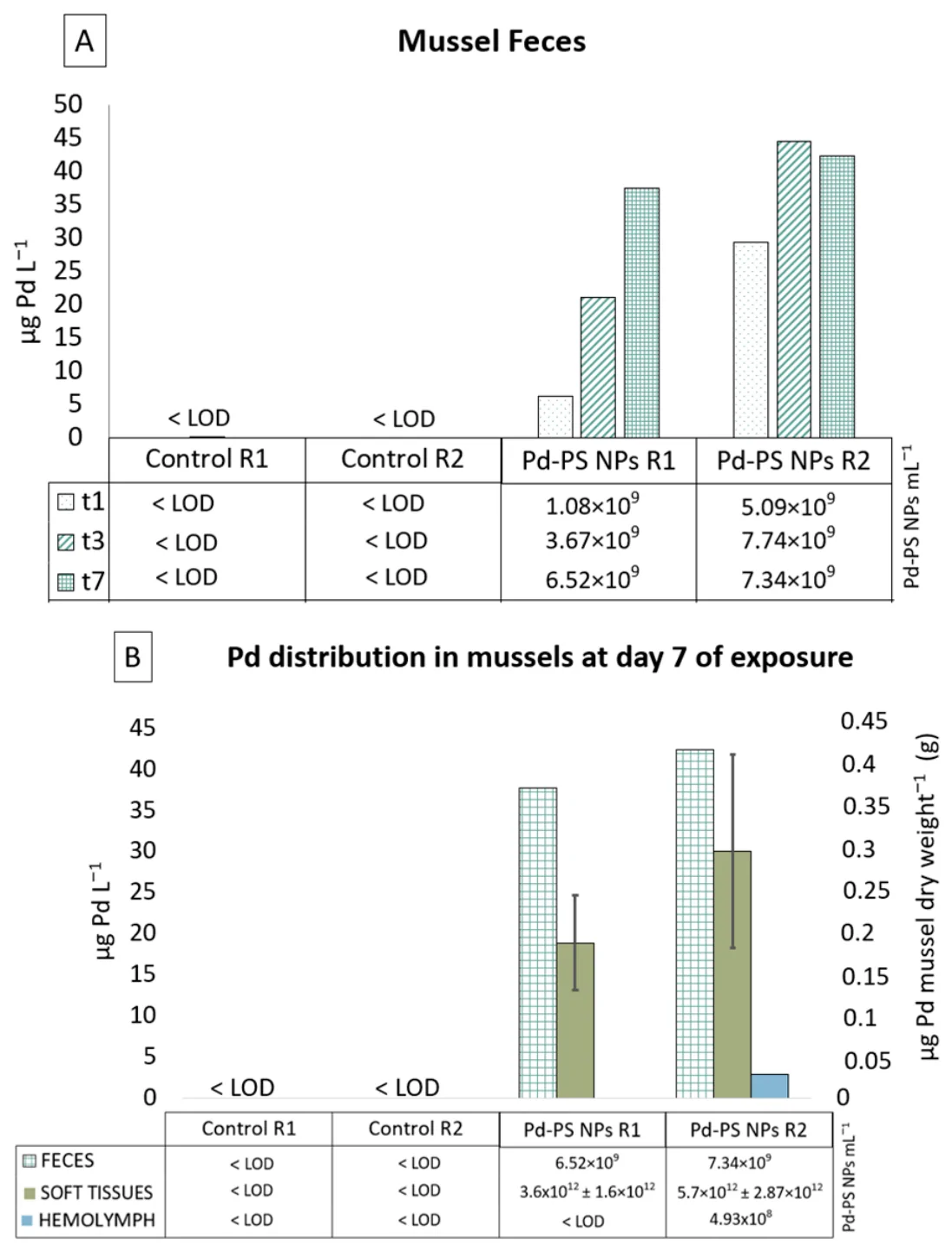
(**A**) Concentration of Pd measured in mussels’ feces (pool of five mussels) over the exposure time (1, 3 and 7 days). (**B**) Concentration of Pd measured in mussels’ feces and hemolymph (left axis) and soft tissues (right axis) at day 7 of exposure. The three measurements correspond to the same mussels. Samples of feces and hemolymph were pooled per tank, whereas the measurement of Pd in soft tissues was performed in each mussel (N = 5 per tank, data given as mean ± SD. Limit of detection (LOD) 0.05 µg L^−1^.

No palladium was detected in feces of mussels from control tanks, while an increase on palladium content was detected over the exposure time in feces of mussels dietarily exposed to Pd-PS NPs; from 17.78 ± 16.35 µg L^−1^ of palladium (3.07 × 10^9^ Pd-PS NP mL^−1^) on day 1 to 39.96 ± 3.33 µg L^−1^ of palladium (6.90 × 10^9^ Pd-PS NP mL^−1^) on day 7 of exposure. The latter corresponded to 14% of the nominal Pd-PS NP exposure on day 7 (Figure 3A).

#### 3.2.4. Mussels’ Hemolymph and Soft Tissues

Similarly, at the end of the exposure, no palladium was detected in the hemolymph and soft tissues of mussels from control tanks (Figure 3B). Meanwhile, palladium was detected in the soft tissues of all analyzed mussels dietarily exposed to Pd-PS NPs, in both replicate tanks (Figure 3B). However, the amount of palladium found in the soft tissues was less than 1% of the nominal Pd-PS NPs dose after 7 days of exposure (Figure 3B). More precisely, a mean of 0.19 ± 0.57 µg of Pd g^−1^ were found in mussels exposed in tank R1 and 0.3 ± 0.12 µg of Pd g^−1^ in mussels exposed in tank R2; which corresponds to 3.6 ± 1.6 × 10^12^ Pd-PS NPs mL^−1^ and 5.7 ± 2.9 × 10^12^ Pd-PS NPs mL^−1^ being present in the tissues of the mussels at day 7 of exposure, respectively (Figure 3B). Similarly, in the case of the hemolymph, palladium was only detectable in the mussels from one replicate tank and it was found in a small amount (≈0.02% of the nominal Pd-PS NPs exposure) (Figure 3B). This value corresponded to 2.84 µg of Pd g^−1^ or 4.93 × 10^8^ Pd-PS NPs mL^−1^ in mussels’ hemolymph (Figure 3B).

#### 3.2.5. Bioaccumulation Factor (BAF) and Mass Balance

After 7 days of foodborne exposure, the BAF of mussels was 43.77 ± 23.09 L kg^−1^, with no significant difference between the mussels of tank R1 (33.80 ± 14.87 L kg^−1^) and tank R2 (57.76 ± 27 L kg^−1^).

**Figure 4:**
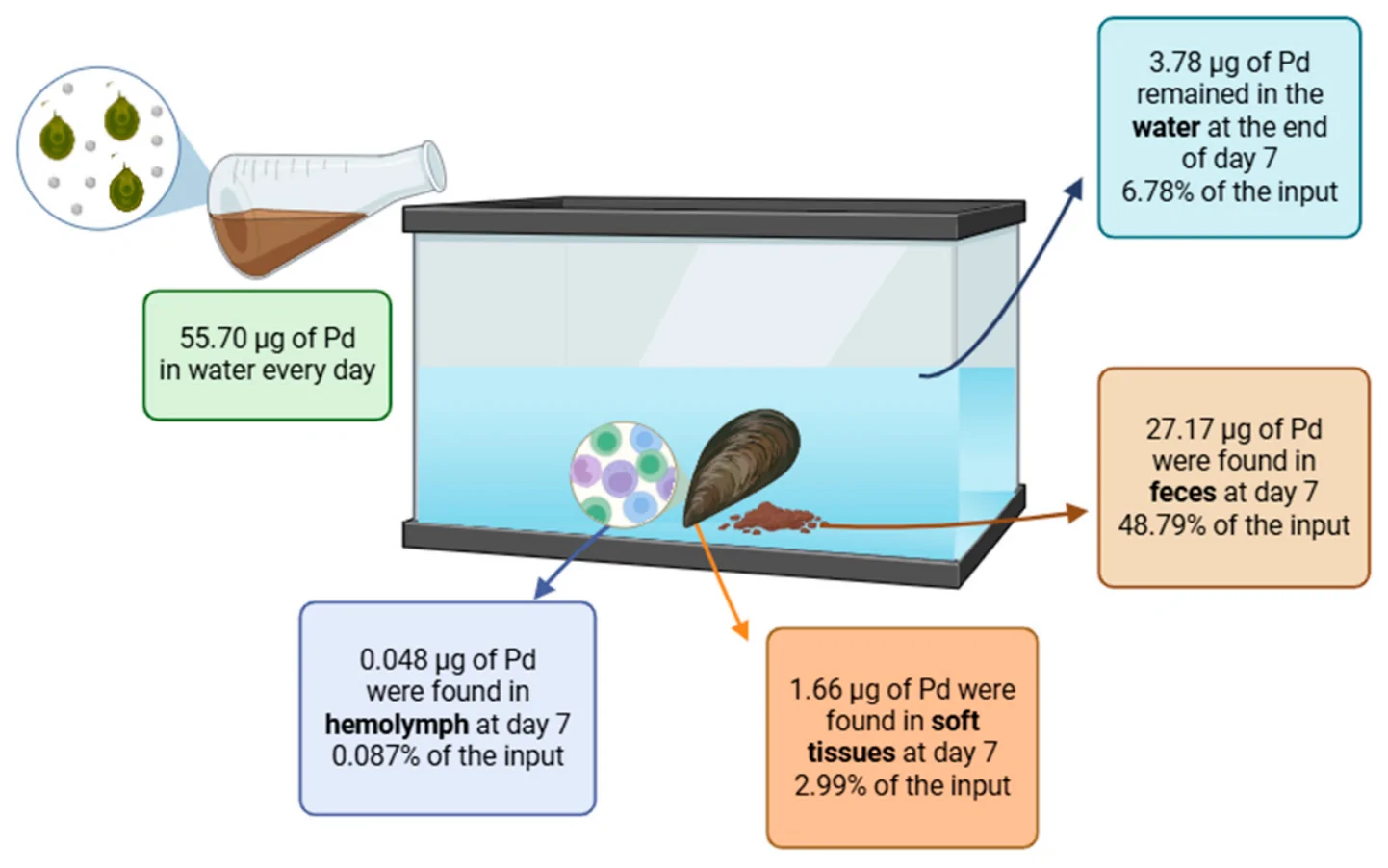
Mass balance at day 7 of exposure, taking into account the daily dose of Pd.

After 7 days of foodborne exposure, 58.65% of the daily Pd dosage was measured (Figure 4). The majority (55.57%) was measured in feces (48.79%) and in water (6.78%). Meanwhile, only 3.1% of the Pd was found in the mussels after 7 days of exposure; 2.99% in the soft tissues and 0.087% in the hemolymph (Figure 4).

### 3.3. Biological Effect Assessment

All mussels were alive and in apparent good health after 7 days of exposure.

#### 3.3.1. Hemocyte Responses

**Figure 5:**
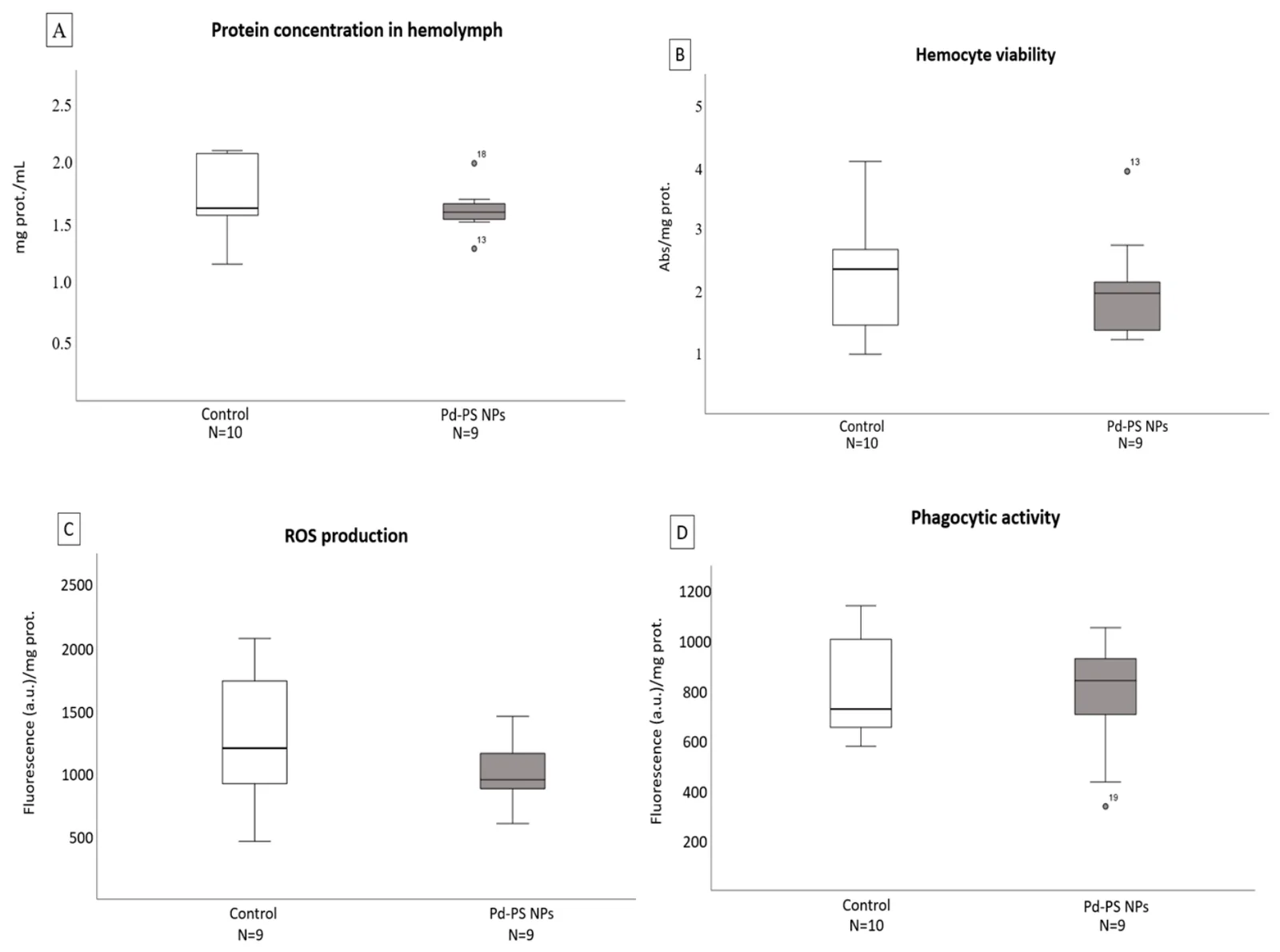
(**A**) Protein concentration in hemolymph; (**B**) Hemocyte viability; (**C**) ROS production; (**D**) Phagocytosis activity of hemocytes of control mussels and mussels exposed to Pd-PS NPs for 7 days. Box plots show median values (horizontal line), 25–75% quartiles (box) and standard deviations (whiskers), considering the two replicates together. Dots denote outliers. No statistically significant differences were observed between groups (*p* > 0.05) according to Mann–Whitney’s U test. *N* is displayed in each graph. “a.u” stands for “arbitrary units”.

No statistically significant differences were observed between control and dietarily exposed mussels in protein concentration of hemolymph (Figure 5A), cell viability (Figure 5B), ROS production (Figure 5C), or phagocytic activity of hemocytes (Figure 5D).

#### 3.3.2. Histopathology of Mussel Digestive Gland

In general, control organisms were nearly free of parasites (Table 2) and showed a good condition of both digestive gland and gonad tissues (Table 2). In the digestive gland, more than half of control mussels showed hemocytic infiltration, a non-specific inflammatory response (Table 2). Other pathologies, such as necrosis or atrophy of digestive tubule epithelium, were sparsely observed in the mussels of the control group (Table 2).

**Figure 6:**
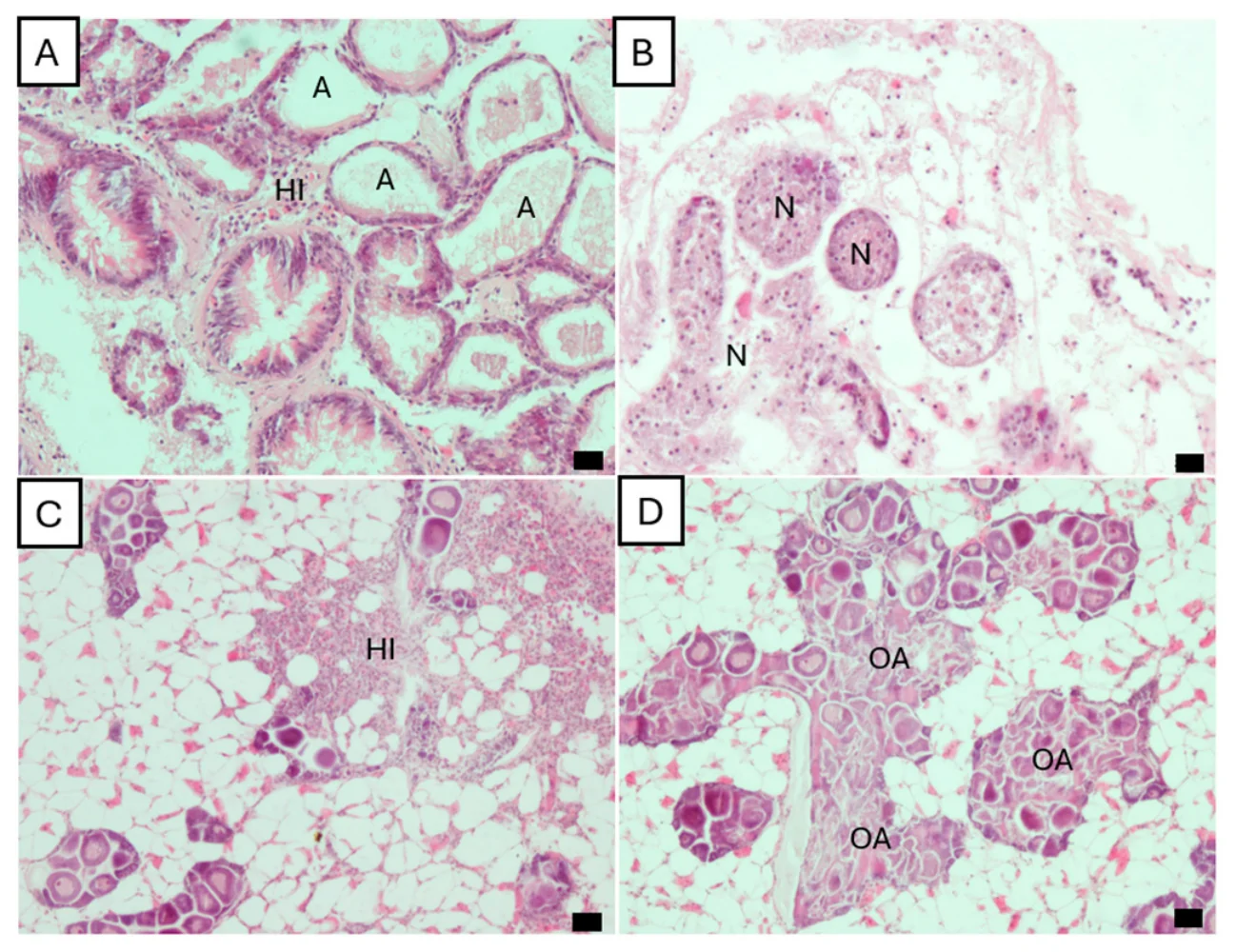
Micrographs of the most common histopathological alterations observed in mussels exposed to Pd-PS NPs. (**A**) Mussels showing atrophy (A) of digestive tubules and hemocytic infiltration (HI) in connective tissue; (**B**) mussels showing necrosis (N) of digestive tubule epithelium; (**C**) mussel showing hemocytic infiltration (HI) in connective tissue of gonad; (**D**) mussel showing oocyte atresia (OA). Scale bars: (**A**,**B**): 20 µm, (**C**,**D**): 10 µm.

In contrast, almost all mussels dietarily exposed to Pd-PS NPs showed digestive tubule atrophy (Figure 6), with a significantly higher intensity in comparison to control mussels (Table 2). In addition, exposed mussels showed a higher prevalence of hemocytic infiltration and fibrosis of connective tissue and necrosis of digestive tubule epithelium than controls (Table 2, Figure 6), but these differences were not statistically significant. In the case of necrosis, the difference was close to significance (*p* = 0.051).

#### 3.3.3. Gamete Development, Gonad Index and Histopathology of Mussel Gonad

**Figure 7:**
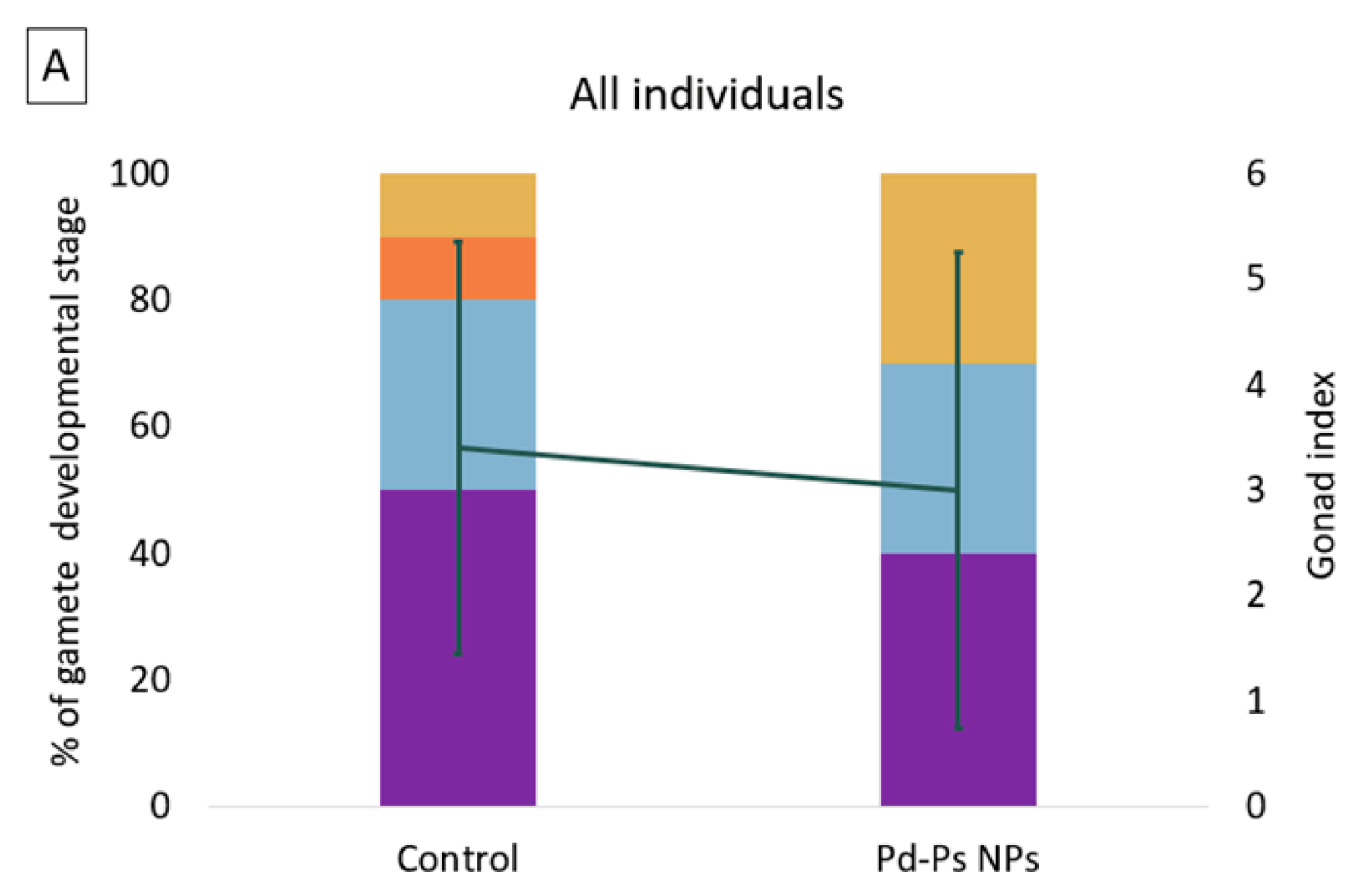

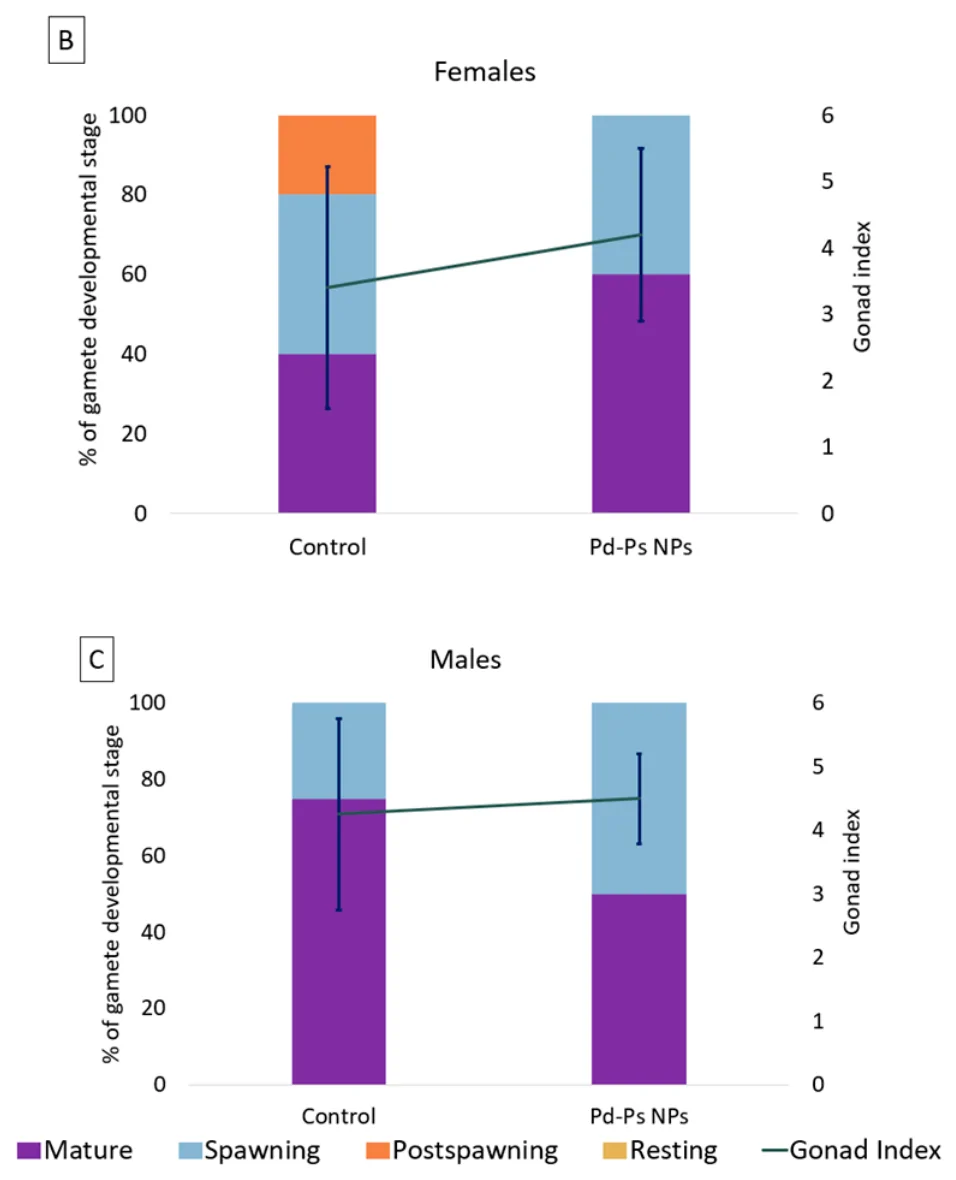
Gonad index (lines) and percentage of mussels at each gamete developmental stage (stacked bars) of control mussels and mussels exposed to Pd-PS NPs for 7 days. (**A**) Both sexes, (**B**) females, and (**C**) males. Gonad index values are given as means and standard deviations. No statistically significant differences were observed between groups (*p* > 0.05) according to Mann–Whitney’s U test.

The sex ratios were 1:1.25 male/female in the control group and 1:2.5 male/female in mussels dietarily exposed to Pd-PS NPs. One and three individuals were classified as undetermined in the control group and in the Pd-PS NPs exposed group, respectively, as these were in the resting gametogenic stage. Thus, sex distribution in control mussels was five females, four males, and one undetermined, whereas in mussels exposed to Pd-PS NPs it was five females, two males, and three undetermined. In general, control mussels were mostly in a mature or spawning gamete developmental stage, while in mussels exposed to Pd-PS NPs, mature was the predominant gamete developmental stage, followed by spawning and resting stages (Figure 7).

When all animals were taken into consideration, the gonad index (GI) was around 3 (Figure 7A). Similar values were observed when only females were taken into consideration for control mussels, but not for exposed mussels, in which GI increased to 4 (Figure 7B). When only males were considered, GI was higher than 4 in both groups (Figure 7C). Overall, there were no significant differences in gamete development and GI between control and exposed mussels.

Collectively, no pathologies were observed in mussel gonads. A slightly higher prevalence of hemocytic infiltration was observed in gonads of exposed mussels (Table 2, Figure 6), but this was not statistically significant. Oocyte atresia was observed in 20% of mussels exposed to Pd-PS NPs (Table 2, Figure 6).

#### 3.3.4. Whole Organism Response: Condition Index

**Figure 8:**
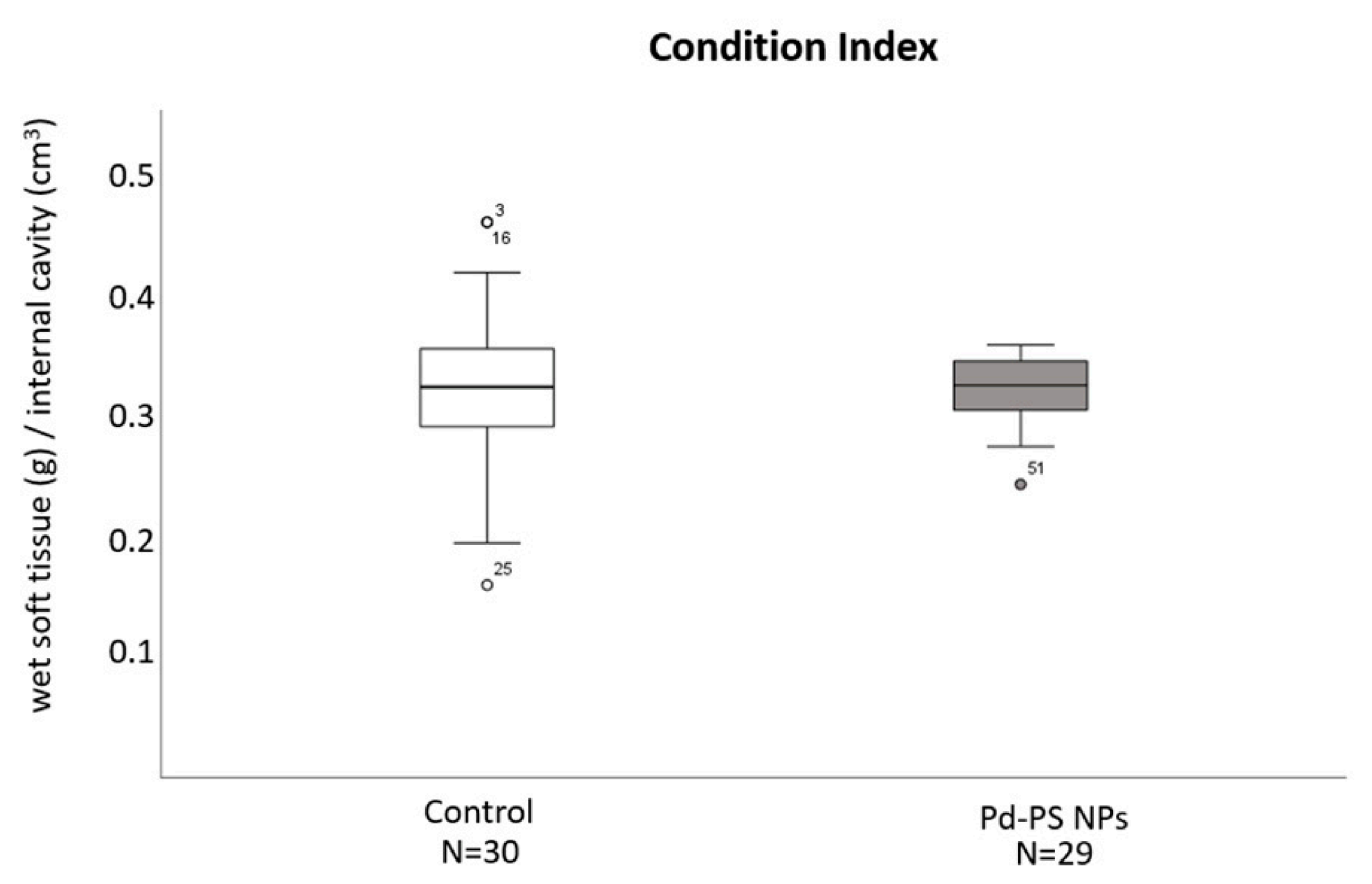
Condition index of control mussels and mussels exposed to Pd-PS NPs for 7 days. Box-plots show median values (horizontal line), 25–75% quartiles (box) and standard deviations (whiskers) considering the two replicates together. Dots denote outliers. No statistically significant differences were observed between groups (*p* > 0.05) according to Mann–Whitney’s U test.

No statistically significant differences were observed between control and dietarily exposed mussels in terms of condition index (Figure 8, Table S3).

## 4. Discussion

Pd-PS NPs tended to aggregate in artificial seawater, increasing their size from 140 nm in ultrapure water up to 300 nm in seawater. Although the stability of Pd-PS NPs is ensured in deionized water in terms of size, concentration, and Z potential [37], previous works have reported aggregation in more complex media such as artificial gut saline solution [41] and natural freshwater from a semi-eutrophic lake [56]. This aggregation trend could favor sinking of Pd-PS NPs proportionally to particle concentration. This would explain the low percentage (47–66%) of Pd-PS NPs recovered in microalgae compared to the nominal exposure dose, in contrast to the high recovery (93%) at t0 of exposure, after pouring microalgae into mussel exposure tanks containing 50 times more water volume.

After the first 24 h of foodborne exposure of mussels, no Pd-PS NPs were detected in exposure water. However, after 7 days of exposure, a concentration higher than 6% (0.378 µg L^−1^) of the daily nominal exposure concentration of Pd-PS NPs was measured in the water. Thus, it can be pointed out that mussels actively take up and remove Pd-PS NPs from the exposure water from the first day of exposure, although a part of them tended to accumulate into tanks water. In addition, the interaction between Pd-PS NPs and mussels could have contributed. It has been reported that when mussels are under non-favorable environmental conditions, including the presence of xenobiotic compounds, they can alter physiological parameters such as clearance rate to accommodate the unfavorable environment [57]. It is well reported in the literature that mussels can indeed reduce their clearance and filtration rates to avoid internalization of undesired compounds into organisms [58,59]. Further, in the case of particulates such as NPs, mussels can increase production of pseudofeces to avoid their internalization [60]. When NPs are incorporated into pseudofeces, these become negatively buoyant, which decreases NPs bioavailability [6]. This could also explain the presence of Pd-PS NPs in water after the last day of exposure.

Several studies have reported internalization of NPs of different sizes in mussels [26,28,61]. The ICP-MS analysis of the present work revealed that after 7 days of exposure, mussels accumulated less than 1% of the nominal exposure concentration of Pd-PS NPs in their soft tissues. Further, less than 0.02% of the Pd-PS NPs were measured in the hemolymph. Similarly, Ribeiro et al. [32] observed some Pd-PS NPs in TEM samples of hemolymph of oysters exposed for 6 days to a concentration of 0.8 mg NPs L^−1^, but Pd concentration was close to LOQ at different exposure times, and therefore, no model could be applied for particle uptake in hemolymph samples [32]. Sendra et al. [26] proved that PS NPs translocated into the hemolymph of mussels in a size-dependent way after an in vivo exposure. PS NPs of 50 nm were observed inside hemocytes, but the 100 nm ones were only observed in the hemolymph serum. This could explain the lack of hemocyte responses observed in the present work, as probably only a few Pd-PS NPs were in contact with cells. Similarly, no detrimental responses were observed in hemocytes after mussels’ in vivo exposure to 500 nm PS NPs in González-Soto et al. [61] or to 1000 nm PS NPs in González-Soto et al. [9]. On the contrary, hemocyte viability decreased when mussels were exposed to 50 nm PS NPs at concentrations as low as 10 µg L^−1^ and 6.87 × 10^−5^ µg L^−1^, respectively [9,28]. However, it must be mentioned that when the exposure is conducted in vitro, mussel hemocytes are able to internalize PS NPs up to 1 µm [27].

The scarce presence of Pd-PS NPs in soft mussel tissues can be explained by the detection of Pd-PS NPs in mussel feces from the first day of exposure. The amount of Pd-PS NPs detected in feces increased during the experiment, with a maximum of 14% of the nominal Pd-PS NPs concentration found in the feces of mussels collected at the end of the experiment. Fernandez & Albentosa [62] observed that mussels eliminated most MPs through feces in less than 24 h post exposure. This would correspond to intestinal feces production time. Brillant & MacDonald [63] showed that bivalves are able to select ingested particles in the stomach based on their size and density. The authors argued that once particles reach the stomach, if those particles are not able to break into smaller pieces by extracellular digestion or if they are too heavy, instead of going into the digestive tubules for intracellular digestion, these can be trapped by the rejection gutter of the stomach and sent to the intestine to be excreted as intestinal feces [63]. Pd-PS NPs used in this study were at the nanoscale, but their density (1.215 g/L) was higher than the density of the usual mussel’s food (a single *I. galbana* cell weight is around 34 pg; thus, the density in the mussel tank was 1.156 mg/L) and could be even increased after aggregation among particles and aggregation with organic material. Thus, it can be hypothesized that a part of the Pd-PS NPs could have been trapped in the sorting areas of the stomach, as proposed by Brillant & MacDonald [63], and these could have been egested in a short period of time. However, the histopathological analysis of the digestive gland, as well as the increase in Pd content in mussel feces over the exposure days, suggested that Pd-PS NPs also followed the glandular path into the digestive ducts to reach the digestive tubules. Some of the Pd-PS NPs could have been egested as glandular feces after intracellular digestion took place, which could have partially impacted the digestive tubules, as was observed in the histological analysis.

The digestive gland is considered the target organ for NP exposure in mussels, as reviewed by Sendra et al. [26]. In bivalves exposed to MPs and NPs, non-specific inflammatory responses are the most commonly reported histopathological alterations [26]; sometimes, the occurrence of necrotic tissue and alterations of digestive tubules have been also reported [61,64,65]. In the present work, mussels exposed to Pd-PS NPs showed a higher prevalence and intensity of digestive tubule atrophy in comparison to controls. In mussels, digestive tubule atrophy is mainly the result of loss of digestive cells, which decreases the height of the epithelium of digestive tubules [47,48]. This condition is often a non-reversible or slowly recoverable stress response [49]. The digestive gland is not only the organ devoted to digestion, but also a key organ for organism homeostasis. Therefore, alterations in the structure of the digestive gland can cause not only failure of the digestive function, but can also have a detrimental impact on nutrient storage, excretion of xenobiotics, and thus, the individual’s physiology [66].

After 7 days of foodborne exposure, the gonad of mussels exposed to Pd-PS NPs barely showed any impact in comparison to controls, probably because the amount of Pd-PS NPs reaching the gonad was limited. Similarly, Ribeiro et al. [32] observed a negligible amount of Pd in the mantle of oysters after 6 days of exposure to Pd-PS NPs. However, tissues such as the mantle in mussels can be indirectly impaired after NPs exposure. In fact, the increased oocyte atresia observed after mussel exposure to MPs has been suggested to represent a mechanism to mobilize energy from oocytes to cope with the increased energy demand [65]. In the present work, an increased prevalence of oocyte atresia was observed in mussels exposed to Pd-PS NPs, but this effect was not statistically significant compared to controls.

According to the mass balance, almost 42% of the Pd-PS NPs measured in water after dosing remained unaccounted for in the analyzed matrices at the end of the experiment. The remaining Pd-PS NPs may have adhered to the tank walls, to the glass beakers used for feces collection, and to the aeration system. Loss via adhesion to the microalgae incubation flask can be discarded, as the measurement done in the mussel water tank at the beginning of the experiment recovered 93% of the dosed Pd-PS NPs. On the other hand, three unanalyzed mussel-associated matrices may account for the missing NPs fraction: the byssus, the shell, and the intervalvar liquid. It has already been reported that MPs can be trapped in mussel byssus, both on the surface and within forming threads [67]. Mussel shells were not analyzed in this study, but it is recommended to analyze them either directly or via surface rinsing/rubbing to recover possible attached Pd-PS NPs. Finally, the intervalvar liquid was not collected for analysis. Analyzing these three mussel-associated matrices and performing a final rinse of all tank equipment at the end of the experiment could improve NP recovery in future studies.

## 5. Conclusions

In conclusion, the Pd-PS NPs applied in this work demonstrated to be a suitable tool to connect the fate and distribution of NPs in mussels with the observed toxicological effects. The results showed that mussels actively removed most of the Pd-PS NPs from water after each foodborne dosing. Mussels were able to expel Pd-PS NPs from the first day of exposure and feces was the matrix where most Pd-PS NPs were detected, with almost 14% at day 7 of exposure. The continuous egestion of Pd-PS NPs could explain why less than 1% of Pd-PS NPs were found in mussels’ soft tissues. The lack of hemocyte responses could be explained by the scarce presence of Pd-PS NPs in the hemolymph. Nevertheless, even if the internalization of the particles appeared to be transitory according to the observation of Pd-PS NPs content in the whole soft tissues, it was enough to cause atrophy of digestive tubules. This suggests that longer exposure times could lead to more severe consequences at the organism level. The trend for increased oocyte atresia and resorption also pointed in that direction. Finally, almost 42% of the Pd-PS NPs measured in water after dosing might have attached to the tanks and associated materials or to mussel byssus and shells. Further work would be necessary to confirm these hypotheses.

## Figures and Tables

**Table 1:** Concentration of Pd measured in the water of mussels’ exposure tanks over the exposure time (t0 and after 1 and 7 days of exposure). Limit of detection (LOD): 0.05 µg L^−1^.

Pd µg L^−1^	t0	t1	t7
Control Tank (R1)	<LOD	<LOD	<LOD
Pd-PS NPs Tank (R1)	5.570	<LOD	0.378

**Table 2:** Prevalence and intensity of histopathological alterations in digestive gland and gonad tissues of control mussels and mussels exposed for 7 days to Pd-PS NPs. The intensity of histopathological alterations is given by an index calculated as the average of the intensities based on a scale from 0 to 4. The asterisk (*) indicates statistically significant differences (*p* < 0.05) between control and exposed mussels according to the *X*^2^ test. N = 10, except for oocyte atresia, where only females were taken into consideration. “n.o.” stands for “not observed”. Representative micrographs for each histopathological alteration can be found in Figure 6.

	Digestive Gland									Gonad		
	Hemocytic Infiltration in the Connective Tissue	Fibrosis	Atrophy of the Digestive Tubules		Necrosis of the Digestive Tubules		Parasites			Hemocytic Infiltration in the Connective Tissue	Oocyte Atresia	
			Prevalence	Intensity (1–4)	Prevalence	Intensity (1–4)	*Mytilicola* sp.	Intracellular Ciliates	Unidentified Protozoa		Prevalence	Intensity (1–4)
Control	60%	10%	20%	1	10%	1	10%	10%	20%	10%	n.o.	0
Pd-PS NPs	90%	20%	90% *	2.22 ± 1.09 *	50%	1	10%	n.o.	n.o.	30%	20%	1

## Data Availability

The raw data supporting the conclusions of this article will be made available by the authors on request.

## Supplementary Materials

The following supporting information can be downloaded at: https://www.mdpi.com/article/10.3390/nano16181133/s1, Table S1: Physical-chemical characterization of Pd-PS NPs. Batch 1 corresponds to the NPs used in the trial experiment and Batch 2 for the ones of the main experiment; Figure S1: Morphology imaging of the Pd-PS NPs using (A) TEM by drop casting method, (B) TEM analysis of resin embedded particles and (C) SEM; Text S1: Trial experiment: mussels exposed to 10^8^ Pd-PS NPs mL^−1^; Table S2: Biometric data of mussels exposed to Pd-PS NPs at the preliminary foodborne experiment; Table S3: Biometric data of mussels exposed to Pd-PS NPs at the main foodborne experiment; Table S4: Biological parameters of control mussels and mussels exposed to Pd-PS NPs per tank.
