# Supplementary material for: Distribution and Effects of Model Palladium-Doped Nanoplastics in the Marine Mussel Mytilus galloprovincialis
Source: Nanomaterials (Basel). 2026 Sep 10;16(18):1133. doi: 10.3390/nano16181133 (PMC13610440; doi:10.3390/nano16181133)
Supplement: Supplementary file 1 [file nanomaterials-16-01133-s001.zip › nanomaterials-4454773-supplementary.pdf]

Supporting Information (SI)

Distribution and effects of model palladium-doped nanoplastics in the marine mussel *Mytilus galloprovincialis*

Nagore González-Soto[1,2], Gabriella F. Schirinzi[3], Guillaume Bucher[3], Eider Bilbao[1], Amaia Orbea[1], Dora Mehn[3], Douglas Gilliland[3], Miguel-Ángel Serra-Beltrán[3&], Denise M. Mitrano[4], Marisa Sárria Pereira de Passos[3‡], Miren P. Cajaraville[1]*

[1]CBET+ Research Group, Department Zoology and Animal Cell Biology, Faculty of Science and Technology and Research Centre for Experimental Marine Biology and Biotechnology PiE, University of the Basque Country UPV/EHU, Basque Country, Spain

[2]EPOC UMR 5805, University of Bordeaux, CNRS, Bordeaux INP, 33600 Pessac, France

[3]European Commission, Joint Research Centre (JRC), Ispra, Italy

[4]Department of Environmental Systems Science, ETH Zurich, Universitätstrasse 16, Zurich, 8092, Switzerland

[&]*current address*: Faculty of Medicine, Universidad Católica San Antonio de Murcia (UCAM), Campus de Murcia, Av. de los Jerónimos, 135, Guadalupe 30107 (Murcia), Spain

[‡]*current address*: Institute of Biotechnology, RWTH Aachen University, Worringerweg 3, 52074 Aachen, Germany

*Corresponding author: mirenp.cajaraville@ehu.eus

Characterization of palladium-doped polystyrene nanoplastics (Pd-PS NPs)

The characterization of Pd-PS NPs involved the determination of various physical-chemical properties. Particle hydrodynamic diameter (size) and polydispersity index (PdI) were measured by Dynamic Light Scattering (DLS). Particle size distribution was analyzed by line start disc centrifugation using a CPS disk centrifuge. Particles net surface charge was measured by Zeta potential. Analytical ultracentrifugation (AUC) was used to determine particles density. Mass concentration of the particles was determined by dry weight, and the Pd content was measured by Inductively Coupled Plasma Mass Spectrometry (ICP-MS).

Table S1. Physical-chemical characterization of Pd-PS NPs. Batch 1 corresponds to the NPs used in the trial experiment and Batch 2 for the ones of the main experiment.

	**Particle size (nm)**		**PdI**	**Net surface charge (mV)**	**Density (g cm^{-3})**	**[Particle mL^{-1}]**	**[Particles] (g L^{-1})**	**[Pd] (mg L^{-1})**
	DLS	*CPS*		at pH 6.59				
Batch 1	223.3 ± 1.62	144.6	0.046 ± 0.025	-35.2 ± 1.56	1.215 ± 0.01	2.6 x 10^{13}	45.09 ± 0.49	114.7 ± 11.2
Batch 2	256.2 ± 13.19	142.6	0.289 ± 0.062	-36.2 ±4.67	1.215 ± 0.01	2.18 x 10^{13}	41.64 ± 0.39	130.2 ± 10.5

Morphology of the Pd-PS NPs was investigated by Transmission Electron Microscopy (TEM) using a JEOL JEM 2100HT microscope (AEMIS User Facilities at the INL Braga, Portugal) coupled to an "OneView" 4k x 4k CCD camera that operates at 25 fps (300 fps with 512 x 512 pixel). SerialEM installed permits low-dose imaging, tomogram acquisition and semi-automation of data collection, making it well suited to characterize both materials and biological samples. Scanning Electron Microscopy (SEM) analysis was further conducted as complement (JRC Ispra, Italy). TEM and SEM micrographs obtained by drop casting permitted to confirm the characteristic raspberry shape of the Pd-PS NPs (Figure S1), as previously reported by Mitrano et al. (2019). Moreover, when embedded in EPON epoxy resin, it was possible to cut the Pd-PS NPs without having these jumping out of the section, which composes a particularly relevant methodological benefit for future TEM analysis of biological samples exposed to the NPs, for which this strategy is notedly suitable. Aside to their characteristic morphology, TEM micrographs permitted us to observe that Pd-PS NPs are more electron dense than EPON epoxy resin, and therefore, easy to identify.

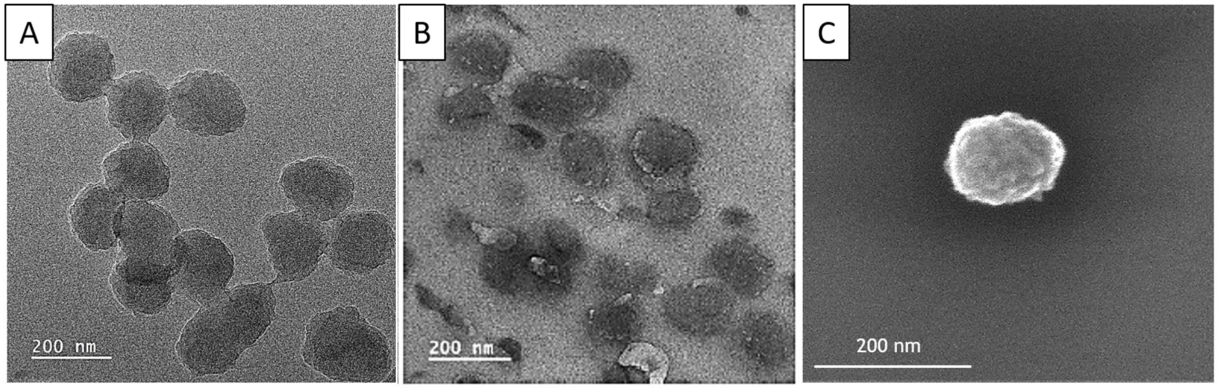

Figure S1. Morphology imaging of the Pd-PS NPs using A) TEM by drop casting method, B) TEM analysis of resin embedded particles and C) SEM.

Text S1: Trial experiment: mussels exposed to 10^8 Pd-PS NPs mL^{-1}

To validate the digestion protocol and determine the limit of detection and quantification of ICP-MS, a small-scale exposure experiment was conducted. *Mytilus galloprovincialis* mussels (3.5-5.5 cm shell length) were sourced from Angulas Aguinaga S.A.U. (Irura, Basque Country, Spain), harvested from Pobra do Caramiñal (42°36'03.8"N, 8°55'34.9"W, Galicia, Spain) in January 2023 (Table S2).

Mussels (n = 5, per tank) were individually placed in 100 mL glass containers, within an 8 L glass tank containing 5 L of filtered seawater. Over a period of 7 days, the mussels were fed daily, after water renewal, with 2 x 10^7 *Isochrysis galbana* cells per mussel. The microalgae were pre-incubated for 24 h with 11.7 x 10^{11} Pd-PS NPs mL^{-1} (2.34 x 10^8 NPs mL^{-1} at the mussel exposure tank). Characteristics of the Pd-PS NPs are given in **Table S1.** In parallel, another 5 mussels were placed in the same conditions in a separate tank and fed with clean *I. galbana* as a control. *I. galbana* was obtained from a clean high-quality microalgae stock from the Spanish National Algae Bank through a commercial dealer and grown at the Plentzia Marine Station (PiE-EHU) microalgae culture room at 19°C, constant light (36 W) and fed with F/2 Algae Food (Fritz Pro Aquatics, Mesquite, Nevada). To ensure the same volume of microalgae/NPs dosage, the microalgae-NPs mixture was prepared daily and taken up to 200 mL with marine water. No mortality was detected during the exposure period. Soft tissue, hemolymph and feces of exposed mussels, as well as microalgae and water samples, were analyzed to determine Pd levels in each matrix.

Sample collection and analysis

Water from the exposure tanks. On the last day of exposure (day 7), 6 replicates of 10 mL of seawater were taken just after redosing (t=0) and at the end of the experiment (t=7). At each time, three of the replicates were immediately frozen and 1 % of HCl was added to the rest before being frozen at -20 °C for long term storage prior further analysis.

Microalgae. An extra incubation of microalgae and Pd-PS NPs was performed, and three aliquots of 20 mL were taken from the 200 mL Erlenmeyer for analysis of Pd content.

Hemolymph and soft tissue of mussels. At the end of the experiment, hemolymph was extracted from the posterior adductor muscle of each mussel and placed in a glass vial with a 1:1 dilution in an anti-aggregant solution (0.47 M NaCl, 1 mM EDTA, pH 7.5). Afterwards, whole mussels' soft tissues were removed from the shell and placed in individual glass vials.

Mussels' feces. Feces of mussels were individually collected from the 100 mL glass containers every day during the 7 days of exposure prior to the water change and redosing. After 7 days of exposure, the feces of each mussel were pooled together.

All biological samples, with the exception of the hemolymph, underwent lyophilization to homogenize and concentrate the samples. Subsequently, these were subjected to acid digestion (HNO_3 ≥ 69 %, Fluka) combining the use of the oxidizing agent H_2O_2 (30 % w/w, Sigma Aldrich), using a microwave digester (CEM Discover SP-D, CEM Corp., Kamp Lintfort, Germany). The following parameters were used: 240 °C, 5 min ramp time, 30 min hold time and 300 W maximal power, followed by a cooling down time of roughly 7 min to 50 °C. The digestion protocol performed was as proposed by Ribeiro et al. (2022) with minor modifications. This digestion method ensures thorough and efficient breakdown of the sample matrix, enabling optimal release and detection of the Pd content by ICP-MS analysis. In the case of hemolymph samples, they were pooled (0.5 mL x 5) and incubated in tubes for 2 h with 0.5 mL of H_2O_2 (30 % w/w, Sigma Aldrich) and 0.4 mL of HNO_3 (≥ 69 %, Fluka) using the Eppendorf ThermoMixer C, set at 80 °C. After cooling, the digested samples were diluted with ultrapure water (18.2 MΩ cm at 25 °C) resulting in a final nitric acid concentration of ~ 5 % (w/w).

The ICP-MS analysis revealed detectable levels of Pd in all matrices except for hemolymph, whether individual or pooled, and the seawater samples. Notably, the Pd levels detected within the microalgae correspond to roughly 49.6 % of the expected nominal mass within the tank, amounting to 1.27 µg Pd L^{-1}. This disparity of values may be attributed to potential interferences from isotopes, particularly from ^{106}Pd originating from ^{88}Sr that is present in seawater (^{88}Sr ^{16}O ^{1}H). Interestingly, Pd concentrations exceeding the limit of quantification (LOQ) were identified in the soft tissues (0.125 ± 0.003 µg Pd g^{-1}) and feces (2.3 µg Pd L^{-1}) of mussels, with no observed interferences during analysis. This suggests that mussels effectively accumulated Pd from the surrounding environment, resulting in measurable concentrations within their soft tissues and fecal matter.

Table S2. Biometric data of mussels exposed to Pd-PS NPs at the preliminary foodborne experiment.

Mussels	Whole wet weight (g)	Shell Weight (g)	Flesh Weight (g)	Length (cm)	Height (cm)	Width (cm)	Internal cavity (cm^3)	Condition Index (wet soft tissue (g) / internal cavity (cm^3))
Control 1	13.89	4.68	9.21	5.2	2.8	1.6	23.29	0.39
Control 2	11.68	4.26	7.42	5.4	2.4	2.2	28.51	0.26
Control 3	14.43	5.29	9.14	6.1	2.9	1.6	28.30	0.32
Control 4	10.37	4.20	6.17	5.3	2.6	1.4	19.29	0.32
Control 5	15.11	5.24	9.87	5.7	2.7	1.6	24.62	0.40
**Pd-PS NPs 1**	16.29	5.83	10.46	5.9	2.7	1.6	25.48	0.41
**Pd-PS NPs 2**	15.49	5.36	10.13	6.0	2.8	1.4	23.52	0.43
**Pd-PS NPs 3**	11.58	3.95	7.63	5.5	2.5	1.3	17.87	0.42
**Pd-PS NPs 4**	16.66	5.94	1.72	5.9	3.0	1.6	28.32	0.37
**Pd-PS NPs 5**	13.93	4.76	9.17	5.6	2.5	1.5	21.00	0.43

Table S3. Biometric data of mussels exposed to Pd-PS NPs at the main foodborne experiment.

Mussels	Whole wet weight (g)	Shell Weight (g)	Flesh Weight (g)	Length (cm)	Height (cm)	Width (cm)	Internal cavity (cm^3)	Condition Index (wet soft tissue (g) / internal cavity (cm^3))
Control R1-1	7.07	2.91	4.16	4.9	1.5	2.9	21.31	0.19
Control R1-2	9.55	2.46	7.08	4.7	1.5	2.6	18.33	0.38
Control R1-3	8.20	1.95	6.25	4.4	1.4	2.2	13.55	0.46
Control R1-4	11.17	2.85	8.32	5.0	1.7	2.6	22.10	0.37
Control R1-5	10.02	2.32	7.70	4.9	1.5	2.5	18.37	0.42
Control R1-6	6.37	2.02	4.35	4.1	1.4	2.2	12.63	0.34
Control R1- 7	10.00	2.43	7.58	5.0	1.6	3.0	24.00	0.31
Control R1-8	5.83	1.57	4.25	4.2	1.4	2.3	13.52	0.31
Control R1-9	7.42	2.21	5.20	4.9	1.5	2.6	19.11	0.27
Control R1-10	8.76	2.76	5.99	4.9	1.5	2.8	20.58	0.29
Control R1-11	10.62	2.95	7.67	5.1	1.6	2.9	23.66	0.32
Control R1-12	7.25	1.96	5.29	4.6	1.4	2.5	16.10	0.33
Control R1-13	9.39	2.55	6.83	5.0	1.7	2.7	22.95	0.29
Control R1-14	9.85	2.71	7.14	4.9	1.7	2.5	20.82	0.34
Control R1-15	8.15	2.26	5.89	4.8	1.5	2.3	16.56	0.35
Control R2-1	9.64	2.17	7.47	4.5	1.5	2.4	16.20	0.46
Control R2-2	8.43	2.94	5.49	4.8	1.7	2.6	21.21	0.26

Control R2-3	6.44	2.54	3.89	4.7	1.6	2.5	18.8	0.21
Control R2-4	10.05	3.45	6.60	5.3	1.7	2.6	23.42	0.28
Control R2-5	8.47	2.79	5.67	5.0	1.7	2.5	21.25	0.27
Control R2-6	7.71	1.98	5.73	4.8	1.6	2.3	17.66	0.32
Control R2- 7	7.67	2.14	5.53	4.9	1.4	2.5	17.15	0.32
Control R2-8	11.66	3.23	8.43	4.7	1.7	2.6	20.77	0.40
Control R2-9	8.72	2.05	6.66	4.7	1.7	2.5	19.97	0.33
Control R2-10	5.35	2.82	2.53	4.7	1.4	2.4	15.79	0.16
Control R2-11	5.74	1.86	3.88	4.1	1.3	2.2	11.72	0.33
Control R2-12	6.73	2.10	4.63	4.6	1.4	2.4	15.45	0.29
Control R2-13	9.76	3.04	6.72	5.0	1.6	2.8	22.40	0.29
Control R2-14	6.36	2.03	4.33	4.4	1.2	2.3	12.14	0.36
Control R2-15	7.70	2.24	5.45	4.7	1.6	2.2	16.54	0.33
**Pd-PS NPs R1-1**	5.67	1.92	3.75	3.9	1.3	2.3	11.66	0.32
**Pd-PS NPs R1-2**	7.54	2.26	5.29	4.3	1.4	2.5	15.05	0.35
**Pd-PS NPs R1-3**	11.59	2.88	8.71	5.1	2.3	2.7	31.67	0.27
**Pd-PS NPs R1-4**	7.69	2.31	5.38	4.6	1.5	2.3	15.87	0.34
**Pd-PS NPs R1-5**	6.32	1.97	4.34	4.3	1.4	2.1	12.64	0.34
**Pd-PS NPs R1-6**	5.34	1.56	3.77	4.0	1.3	2.1	10.92	0.34
**Pd-PS NPs R1-7**	9.71	2.74	6.96	4.9	1.7	2.7	22.49	0.31

**Pd-PS NPs R1-8**	8.11	2.42	5.69	4.4	1.5	2.4	15.84	0.36
**Pd-PS NPs R1-9**	6.69	2.01	4.68	4.4	1.4	2.4	14.78	0.32
**Pd-PS NPs R1-10**	7.87	2.20	5.67	4.7	1.5	2.7	19.03	0.30
**Pd-PS NPs R1-11**	7.90	2.64	5.26	4.6	1.5	2.5	17.25	0.30
**Pd-PS NPs R1-12**	8.00	2.64	5.37	4.3	1.7	2.4	17.54	0.30
**Pd-PS NPs R1-13**	8.17	2.45	5.72	4.9	1.5	2.3	16.90	0.34
**Pd-PS NPs R1-14**	9.73	2.84	6.89	5.0	1.1	2.5	13.75	0.50
**Pd-PS NPs R1-15**	9.01	2.46	6.55	4.7	1.9	2.5	22.32	0.29
**Pd-PS NPs R2-1**	6.37	1.77	4.60	4.2	1.4	2.2	12.94	0.35
**Pd-PS NPs R2-2**	6.74	2.23	4.51	4.7	1.4	2.4	15.79	0.28
**Pd-PS NPs R2-3**	9.65	2.78	6.88	4.6	1.7	2.7	21.11	0.32
**Pd-PS NPs R2-4**	10.20	2.92	7.28	5.0	1.7	2.7	22.95	0.32
**Pd-PS NPs R2-5**	8.09	2.42	5.68	4.7	1.6	2.3	17.30	0.33
**Pd-PS NPs R2-6**	7.68	2.41	5.27	4.9	1.7	2.6	21.66	0.24
**Pd-PS NPs R2-7**	9.07	2.57	6.50	4.9	1.6	2.7	21.17	0.31
**Pd-PS NPs R2-8**	5.90	1.83	4.07	4.2	1.3	2.5	13.65	0.30
**Pd-PS NPs R2-9**	9.48	2.45	7.03	4.9	1.7	2.9	24.16	0.29

**Pd-PS NPs R2-10**	7.34	1.97	5.38	4.5	1.5	2.3	15.52	0.34
**Pd-PS NPs R2-11**	6.41	1.74	4.66	4.5	1.4	2.1	13.23	0.35
**Pd-PS NPs R2-12**	8.40	2.28	6.12	4.8	1.5	2.4	17.28	0.35
**Pd-PS NPs R2-13**	7.74	2.30	5.44	4.5	1.4	2.5	15.75	0.34
**Pd-PS NPs R2-14**	8.76	2.66	6.09	4.8	1.5	2.6	18.72	0.32
**Pd-PS NPs R2-15**	8.10	2.39	5.71	4.7	1.4	2.5	16.45	0.35

Table S4. Biological parameters of control mussels and mussels exposed to Pd-PS NPs per tank. n.o. not observed.

	HEMOCYTE RESPONSES				GONAD INDEX			CONDITION INDEX (wet soft tissue (g) / internal cavity (cm^3))
	Protein concentration of hemolymph (mg/ml)	Cell viability (MTT) (abs/mg prot.)	ROS (fluo./mg prot.)	Phagocytic activity (fluo./mg prot.)	All individuals	Only males	Only females	
Control R1	1.88 ± 0.28	2.17 ± 0.98	1285.98 ± 672.78	723.86 ± 174.62	4 ± 1.73	5 ± 0	3.33 ± 2.08	0.35 ± 0.05
Control R2	1.47 ± 0.21	2.41 ± 1.07	1236.57 ± 301.04	863.41 ± 206.85	2.80 ± 2.17	3.5 ± 2.12	3.50 ± 2.12	0.31 ± 0.07
Pd-PS NPs R1	1.55 ± 0.05	1.99 ± 0.63	964.96 ± 211.92	853.53 ± 17.33	3.20 ± 2.17	5 ± 0	3.67 ± 1.53	0.32 ± 0.02
Pd-PS NPs R2	1.88 ± 0.38	1.65 ± 0.37	1307.88 ± 886.15	684.18 ± 276.54	2.80 ± 2.59	2.80 ± 2.59	2.80 ± 2.59	0.33 ± 0.02

	HISTOPATHOLOGY											
	Digestive gland									Gonad		
	Hemocytic infiltration in the connective tissue	Fibrosis	Atrophy of the digestive tubules		Necrosis of the digestive tubules		Parasites			Hemocytic infiltration in the connective tissue	Oocyte atresia	
			Prevalence	Intensity (1-4)	Prevalence	Intensity (1-4)	*Mytilicola sp.*	Intracellular ciliates	Unidentified protozoa		Prevalence	Intensity (1-4)
Control R1	60%	n.o.	n.o.	n.o.	n.o.	n.o.	20%	20%	20%	20%	n.o.	n.o.
Control R2	60%	20%	40%	1 ± 0	20%	1 ± 0	n.o.	n.o.	20%	n.o.	n.o.	1 ± 0
Pd-PS NPs R1	100%	40%	100%	2.2 ± 1.30	60%	1 ± 0	n.o.	n.o.	n.o.	40%	20%	1 ± 0
Pd-PS NPs R2	80%	n.o.	80%	2.25 ± 0.95	40%	1 ± 0	20%	n.o.	n.o.	20%	20$	1 ± 0
